# Draft Whole-Genome Sequences of Campylobacter Strains Isolated from Brushtail Possums in New Zealand

**DOI:** 10.1128/MRA.01276-19

**Published:** 2019-11-21

**Authors:** David A. Wilkinson, Lynn E. Rogers, Ahmed Fayaz, Rukhshana N. Akhter, Patrick J. Biggs, Nigel P. French, Anne C. Midwinter

**Affiliations:** a ^m^EpiLab, Infectious Disease Research Centre, School of Veterinary Science, Massey University, Palmerston North, New Zealand; b New Zealand Food Safety Science and Research Centre, Massey University, Palmerston North, New Zealand; University of Maryland School of Medicine

## Abstract

Draft genomes of five Campylobacter isolates recovered from New Zealand brushtail possums are described. Genome sizes ranged from 1.591 Mbp to 1.594 Mbp, with G+C contents of 29.9% to 29.95%. Comparison to Australian *Campylobacter* 16S rRNA gene sequences suggests that the species may be common to possums.

## ANNOUNCEMENT

In the 1850s (1), the Australian brushtail possum (Trichosurus vulpecula; Maori, *paihamu*) was first introduced to New Zealand, where it rapidly became a significant invasive pest (1). Although Campylobacter spp. have been described in the Australian brushtail possum population (2, 3), they have not previously been isolated from the New Zealand population (4, 5). We hypothesized that brushtail possums play a role in the epidemiology of *Campylobacter* spp. in New Zealand, contributing to the contamination of waterways and other environments (6).

Swabs from the cecum, intestine, or feces were taken from road-killed possums or possums killed as routine pest control in an urban or perirural setting (Palmerston North, New Zealand). Swabs were cultured on cefoperazone amphotericin teicoplanin (CAT) agar (Fort Richard Laboratories, Auckland, New Zealand) in an H_2_-enriched microaerobic atmosphere at 37°C, and colonies typical of *Campylobacter* spp. were seen after 3 days. Single colonies were subcultured on Columbia horse blood agar (Fort Richard Laboratories) and grown under the same conditions for genomic DNA preparation. Genomic DNA was extracted using a QIAamp DNA minikit (Qiagen, Hilden, Germany). DNA was checked for quality using Qubit assay kits (Life Technologies, Oregon, USA) and for fragmentation using gel electrophoresis. Genomic DNA was sequenced at New Zealand Genomics, Ltd. (Massey University, Palmerston North, New Zealand), using either an Illumina MiSeq or Illumina HiSeq 2500 instrument (Scoresby, Victoria, Australia) according to the manufacturer’s instructions with paired-read lengths of 250 and 150 bp, respectively. Sequence data were trimmed using Trimmomatic v.0.3.8 (7) (trim parameters, 1:30:11 LEADING:10 TRAILING:10 MINLEN:30), assembled using SKESA v.2.2.1 (8) using the default settings, and further processed and annotated online by the NCBI Prokaryotic Genome Annotation Pipeline (9). Relevant sequencing, assembly, and genome statistics are described in Table 1.

**TABLE 1:** *Campylobacter* sp. genome statistics

Strain	Length (bp)	No. of contigs	*N*_50_ (bp)	Coverage (×)	No. of CDSs ^*a*^	G+C content (%)	NCBI assembly accession no.	GenBank accession no.	SRA accession no.	Sequence type	Raw sequences (Mbp)
LR185c	1,591,228	55	64,039	101	1,659	29.95	GCA_008633905	VJNR00000000	SRR9678926	MiSeq 2 × 250 bp	160.4
LR196d	1,592,443	41	117,332	463	1,665	29.91	GCA_008633865	VJNS00000000	SRR9678927	HiSeq 2 × 150 bp	736.9
LR264d	1,593,663	40	129,728	381	1,671	29.9	GCA_008633895	VJNT00000000	SRR9678928	HiSeq 2 × 150 bp	606.6
LR286c	1,592,793	47	78,517	414	1,671	29.92	GCA_008633875	VJNU00000000	SRR9678929	HiSeq 2 × 150 bp	659.2
LR291e	1,594,282	39	129,696	455	1,668	29.92	GCA_008633915	VJNV00000000	SRR9678925	HiSeq 2 × 150 bp	725.7

a CDSs, coding DNA sequences.

Genomes ranged in size from 1,591,228 bp to 1,594,282 bp with between 1,659 and 1,671 predicted coding sequences. The G+C contents were between 29.9% and 29.95%. All genomes had single copies of 5S, 16S, and 23S rRNA gene sequences and 33 identifiable tRNAs. The 16S rRNA sequences from all isolates were identical and showed the closest BLAST similarity to *Campylobacter* isolate BTP1Tcr (GenBank accession number AY554142), with a pairwise sequence identity of 99.6% over 1,427 bp. This sequence was obtained from a study of Australian brushtail possums (2), which identified both Helicobacter and *Campylobacter* carriage. This species of *Campylobacter* possibly forms an association with the gastrointestinal tract of possums. Comparison with representative 16S rRNA sequences from all other *Campylobacter* species showed that the closest similarity was to Campylobacter helveticus (98.7% identity, NCBI assembly accession numbers GCF_002080395 and GCF_900176295), with similar observed levels of identity with Campylobacter upsaliensis (98% to 98.4% identity, GCA_000167395, GCA_000185345, and GCA_000620965) and Campylobacter avium (98.2% identity, GCA_002238335 and GCA_002245935).

### Data availability.

*Campylobacter* genomes from this article are submitted under BioProject accession number PRJNA552733 and BioSample accession numbers SAMN12216776 through SAMN12216780, with GenBank accession numbers VJNR00000000, VJNS00000000, VJNT00000000, VJNU00000000, and VJNV00000000, corresponding to SRA accession numbers SRR9678926, SRR9678927, SRR9678928, SRR9678929, and SRR9678925, respectively.
